# *COMT, 5-HTR2A*, and *SLC6A4* mRNA Expressions in First-Episode Antipsychotic-Naïve Schizophrenia and Association With Treatment Outcomes

**DOI:** 10.3389/fpsyt.2018.00577

**Published:** 2018-11-13

**Authors:** Zongchang Li, Ying He, Hongying Han, Yao Zhou, Xiaoqian Ma, Dong Wang, Jun Zhou, Honghong Ren, Liu Yuan, Jinsong Tang, Xiaofen Zong, Maolin Hu, Xiaogang Chen

**Affiliations:** ^1^Mental Health Institute of the Second Xiangya Hospital, Central South University, Changsha, China; ^2^Chinese National Clinical Research Center on Mental Disorders, Chinese National Technology Institute on Mental Disorders, Hunan Key Laboratory of Psychiatry and Mental Health, Changsha, China; ^3^Center for Medical Genetics, School of Life Sciences, Central South University, Changsha, China; ^4^Department of Psychiatry, The Third Affiliated Hospital of Sun Yat-sen University, Guangzhou, China; ^5^Wuxi Mental Health Center, Nanjing Medical University, Wuxi, China; ^6^Department of Psychiatry, Renmin Hospital of Wuhan University, Wuhan, China

**Keywords:** schizophrenia, *COMT*, *5-HTR2A*, *SLC6A4*, antipsychotic response, cognitive deficits

## Abstract

**Background:** Dopaminergic and serotonergic systems play crucial roles in the pathophysiology of schizophrenia and modulate response to antipsychotic treatment. However, previous studies of dopaminergic and serotonergic genes expression are sparse, and their results have been inconsistent. In this longitudinal study, we aim to investigate the expressions of Catechol-O-methyltransferase (*COMT*), serotonin 2A receptor (*5-HTR2A*), and serotonin transporter gene (*SLC6A4*) mRNA in first-episode antipsychotic-naïve schizophrenia and to test if these mRNA expressions are associated with cognitive deficits and treatment outcomes or not.

**Method:** We measured *COMT, 5-HTR2A*, and *SLC6A4* mRNA expressions in 45 drug-naive first-episode schizophrenia patients and 38 health controls at baseline, and repeated mRNA measurements in all patients at the 8-week follow up. Furthermore, we also assessed antipsychotic response and cognitive improvement after 8 weeks of risperidone monotherapy.

**Results:** Patients were divided into responders (*N* = 20) and non-responders groups (*N* = 25) according to the Remission criteria of the Schizophrenia Working Group. Both patient groups have significantly higher *COMT* mRNA expression and lower *SLC6A4* mRNA expression when compared with healthy controls. Interestingly, responder patients have significantly higher levels of *COMT* and *5-HTR2A* mRNA expressions than non-responder patients at baseline. However, antipsychotic treatment has no significant effect on the expressions of *COMT, 5-HTR2A*, and *SLC6A4* mRNA over 8-week follow up.

**Conclusion:** Our findings suggest that dysregulated *COMT* and *SLC6A4* mRNA expressions may implicate in the pathophysiology of schizophrenia, and that *COMT* and *5-HTR2A* mRNA may be potential biomarkers to predict antipsychotic response.

## Introduction

Schizophrenia is a complex and devastating psychiatric disorder affecting ~1% of the population. It is characterized by a set of psychotic symptoms such as hallucinations, delusions, cognitive impairments, and negative symptoms (1). Despite substantial efforts in recent decades, the pathophysiology of schizophrenia remains poorly delineated. So far, antipsychotic drugs are still the primary pharmacological treatment of schizophrenia. However, ~30% of the patients have not received satisfied symptomatic improvement after accepting antipsychotic medications with an adequate dosage and duration mainly due to the long-term morbidity and poor functional outcome in schizophrenia (2). Consequently, identifying reliable biomarkers for antipsychotic response has been considered as a promising strategy to improve the therapeutic effects to individual patients with the existing antipsychotic drugs.

Dysfunction in neurotransmitter systems is a well-established pathological feature of schizophrenia. Multiple lines of evidence indicated that dopaminergic and serotonergic systems and their interactions play critical roles in the mechanistic underpinnings as well as the antipsychotic response in schizophrenia (3–5). First, molecular imaging studies have reported the dysregulation of dopamine and neurotransmission in the striatum and prefrontal cortex (PFC) (3, 6–8), which is critical for cognition and emotional processing that has been implicated in the pathogenesis of schizophrenia. Second, cerebral dopamine and serotine receptor systems are the main targets of antipsychotic medications in treating schizophrenia. For example, the most effective atypical antipsychotics such as risperidone, olanzapine, and clozapine have the highest affinity to *DAD2* and *5-HT2A* receptors (9, 10). Moreover, the serotonin-dopamine interactions that modulate prefrontal brain activity have also received extensive focus as the development of treatment of schizophrenia (11, 12). Finally, drugs that drive dopamine/serotine release or increase dopamine/serotine transmission, such as amphetamine and lysergic acid diethylamide (LSD) will exacerbate psychosis in patients with schizophrenia and also can induce schizophrenic symptoms in healthy individuals at high doses (13, 14).

Candidate genetic association studies of dopaminergic and serotoninergic genes for schizophrenia and antipsychotic response have been widely conducted. Four candidate genes (DRD2, COMT, 5-HTR2A, and SLC6A4) from dopaminergic and serotoninergic pathways have received the most attention in the literature and have been supposed as the promising candidate genes for schizophrenia and treatment response (15–19). However, only DRD2 gene has been consistently replicated in studies with other strategies. For example, GWAS of schizophrenia found that variant of *DRD2* is significantly associated with schizophrenia at the genome-wide significance level (20), and genetic drug-target networks analyses supported that DRD2 gene might play important and regulatory roles in the biological pathway of antipsychotic treatment (21–23). It is unsurprising for the limited successes in genetic association studies of schizophrenia owing to the heterogeneous nature of symptomatology and the small effect sizes of individual variants on phenotypic traits.

Recent evidence has shown that numerous GWAS significant risk variants locate in the non-coding regions and are enriched for expression quantitative trait locus (eQTLs) (20). In this regard, the application of gene expression analysis may provide an opportunity to investigate the pathogeneses of complicated clinical diseases such as schizophrenia. Many previous expression researches have reported that dysregulated expression of dopaminergic and serotoninergic genes in brain may implicated in the pathogenesis of schizophrenia (24–27). Furthermore, experimental evidences supported that antipsychotic treatment can regulate dopaminergic and serotoninergic genes expressions, implicating these genes might play roles in symptom improvement over antipsychotic treatment (28–30). Recently, many findings have indicated that peripheral gene expression may provide useful information on aberrant neurotransmission in neuropsychiatric diseases and monitor the drug response despite several methodological and theoretical limitations (31–33). Based on the above evidences, the present study measured *COMT, 5-HTR2A*, and *SLC6A4* mRNA expressions in first-episode antipsychotic-naïve schizophrenia and followed up for their treatment response after 8 weeks' risperidone monotherapy. These three candidate genes were selected in our study because they have been supposed to be the most prominent in relation to disease pathophysiology and antipsychotic treatment but have been considerably inconsistent. The aims of this study are to explore (1) whether *COMT, 5-HTR2A*, and *SLC6A4* mRNA expressions are altered in schizophrenia patients; (2) whether *COMT, 5-HTR2A*, and *SLC6A4* mRNA expressions are associated with treatment outcomes, cognitive defects and psychopathological symptoms in schizophrenia; (3) whether antipsychotic treatment has effect on *COMT, 5-HTR2A*, and *SLC6A4* mRNA expressions.

## Materials and methods

### Subjects

Forty-five first-episode antipsychotic-naïve schizophrenia patients and thirty-eight age-matched controls were recruited in this study. Individuals were diagnosed with first-episode schizophrenia by experienced psychiatrists using the Structured Clinical Interview for DSM IV Disorders, and none of the patients was ever treated with antipsychotic medications or other psychotropics before. Psychotic symptoms severity was evaluated using the 30-item Positive and Negative Symptom Scale (PANSS) and only patients with PANSS score ≥65 were selected. Patients with any other DSM-IV diagnoses or clinically significant medical diseases were excluded. Healthy controls were screened using the SCID-NP (Non-Patient Edition) and were recruited if they are absent of any psychiatric disorder for the lifetime. Inclusion criteria for all participants were aged between 18 and 40 years, being right handed and having normal vision. The study design and procedures were in accordance with the Declaration of Helsinki, and approved by The Ethics Committee of the Second Xiangya Hospital of Central South University. All participants gave written informed consent after the procedures had been fully explained to participation in the study.

### Clinical assessment and treatment response

All patients were treated with risperidone monotherapy for 8 weeks. The dosage of risperidone was 2 mg per day initially and then was adjusted by the attending psychiatrist according to individual tolerance. For all patients, risperidone compliance was closely monitored by clinical interviews, and no mood stabilizers and antidepressants were given. Psychotic symptoms at baseline and after 8-week of treatment were evaluated using PANSS.

Treatment response at 8 weeks was evaluated with the Remission criteria of the Schizophrenia Working Group (RSWG) as the primary outcome (34). The criteria identifies an absolute threshold in the severity of symptoms and defines patients as responders if they simultaneously had a final score ≤ 3 for at least 6 months on eight core symptoms of the PANSS: P1, P2, P3, N1, N4, N6, G5, and G9. This study used the criteria without the duration requirement.

### Cognitive assessments

Cognitive assessments were conducted by two well-trained psychiatrists. The cognitive domains were selected for executive function, verbal fluency, attention, and processing speed, attention distribution, working memory, and motor speed. We assessed cognitive function using Wisconsin Card Sorting Test (WCST-C: categories and WCST-P: perseverative errors, 128 cards), Verbal Fluency Test (VFT), Trail Making Test (TMT-part A and TMT-part B), Digit Span Tests (DST-Forward and DST-Backward), and Stroop Tests (Stroop-W: words, Stroop-C: colors, and Stroop-I: interference). The details of cognitive assessments have been described elsewhere (35). All participants were assessed cognitive function at baseline and 36 patients repeated cognitive assessments after 8-week follow-up. Cognitive improvement was measured by using the difference of cognitive performance between follow-up and baseline.

### Expression analyses

Blood was collected from all participants in BD tubes before being processed. Total RNA sample was isolated by using the TRIZOL Reagent (Life Technologies). The RNA integrity was determined through electrophoresis on a 1.0 % agarose gel. The concentrations and purity of the RNA samples were determined using the Nanodrop 2000 spectrophotometer (Thermo Scientific, Wilmington, DE, USA). Two micrograms of total RNA for each sample was used for complementary DNA (cDNA) synthesis using the High-Capacity cDNA Reverse Transcription Kit (Applied Biosystems, CA, USA) in a total reaction volume of 20 μL, according to the manufacturer's protocol.

The mRNA expression of *COMT, 5-HTR2A*, and *SLC6A4* were measured by quantitative polymerase chain reaction (qPCR). We designed different pairs of primers and probes to detect the targeting mRNA expression. The primers for *COMT* mRNA expression are: forward: 5′- TGGTACTGAAGGTGCCAGAC-3′; reverse: 5′-GTTCAGAGAGGTTAGCATGTCA-3′; probe: FAM-CCTGCTGACCTTCTGCGGCTCC-BHQ1. The primers for *5-HTR2A* mRNA expression are: forward: 5′-AATAGCGACGGAGTGAATGA-3′; reverse: 5′-AGATAGGTGAAAACTTGCTCAG-3′; probe: FAM-TGCCTTCCACAGTTGCCACGG-BHQ1. The primers for *SLC6A4* mRNA expression are: forward: 5′-GATGAGTTCCCACACGTCTG-3′; reverse: 5′-GTGACCAGGGATCCAAAGAAG-3′; probe: FAM-TGACCACGGCGAGCACGAACC-BHQ1. We selected the housekeeping gene glyceraldehyde-3-phosphate dehydrogenase (*GAPDH*) as endogenous controls, because it is adequate expression in the blood and shows high stability between the different samples (36, 37). The *GAPDH* primers: forward: 5′-GTTCCAATATGATTCCACCCATG-3′; reverse: 5′-GGGATTTCCATTGATGACAAGC-3′; probe: FAM-CATGGCACCGTCAAGGCTGAGAAC-BHQ1. All qPCR were performed with TaqMan Universal PCR Master Mix on a Roche LightCycler® 480 machine (Roche Applied Science, Mannhein, Germany). Each sample was quantified in triplicate with an initial denaturing step of 10 min at 95°C, followed by 40 cycles of 15 s at 95°C and 60 s at 60°C. The efficiencies of quantitative PCR amplifications for both target and reference genes were tested by a wide range of diluted cDNA dilution and ranges from 92 to 102%. Relative mRNA expression was calculated with comparative Ct method (ΔCt = Ct target–Ct reference; Supplementary file), and data were analyzed using the Light cycler® 480 software Version 1.5.

### Data analyses

All data were analyzed using the Statistical Package for Social Sciences (SPSS, version 20.0 for Windows). Kolmogorov-Smirnov tests were performed to detect the normal distribution of continuous variables, and data with skewed distribution were natural transformed prior to analysis. Data in schizophrenia patients (responders and non-responders) and controls were compared using chi-squared test for categorical variables, and independent Student's *t*-test or one-way ANOVA followed by the least significant difference (LSD) *post-hoc* analyses were applied for continuous variables. To examine the interaction between *COMT, 5-HTR2A*, and *SLC6A4* mRNA expressions, partial correlation analyses between these three genes were tested in the patient and control groups separately controlling for age, gender and education. Furthermore, exploratory regression analyses were performed to examine the relationships of these three genes expressions with baseline clinical phenotypes (symptom severity and cognition) as well as cognitive improvement after treatment in patients. Correlations between baseline mRNA expression of *COMT* (or *5-HTR2A, SLC6A4*) and clinical variables (baseline total PANSS score, baseline cognitive scores, or cognitive improvement) were computed by Stepwise multiple regression analyses, controlling for age, gender, education, and duration of illness. Bonferroni corrections were conducted to each test to adjust for multiple testing. Finally, we also performed repeated measures ANOVAs (RMANOVAs) to test longitudinal within-group changes (responders and non-responders), and group × time (baseline and follow-up) interaction for the levels of mRNA expression. All analyses were two-sided and *p*-value of 0.05 was considered statistically significant.

## Results

### Demographic data

Demographic and clinical characteristics of all participants were shown in Table 1. According to RSWG criteria, 20 patients were classified as responders and 25 patients were classified as non-responders. There are no significant differences in age, sex, and education among three groups (Table 1). No significant differences of symptom severities at baseline between responders and no-responders in PANSS total symptoms (91.45 ± 15.80 vs. 92.68 ± 10.50, *p* = 0.756), PANSS positive symptoms (25.45 ± 4.47 vs. 25.44 ± 3.85, *p* = 0.994), PANSS negative symptoms (19.30 ± 6.18 vs. 18.58 ± 5.59, *p* = 0.726), and PANSS general symptoms (46.70 ± 8.18 vs. 48.56 ± 6.49, *p* = 0.400) were observed.

**Table 1:** Demographic and clinical characteristics in full sample at baseline.

	**Responders**	**No-responders**	**Controls**	**Test and signifcance**
Number	20	25	38
Sex/Female	6	8	13	χ = 0.11, *p* = 0.946
Age	26.3 ± 5.01	23.04 ± 4.15	24.76 ± 4.55	*F* = 2.83, *p* = 0.065
Education/years	10.70 ± 4.35	9.84 ± 2.95	11.05 ± 2.91	*F* = 1.25, *p* = 0.291
Illness duration /months	8.50 ± 2.78	8.64 ± 2.80	–	*t* = −0.17, *p* = 0.868
PANSS-P	25.45 ± 4.47	25.44 ± 3.85	–	*t* = 0.01, *p* = 0.994
PANSS-N	19.30 ± 6.18	18.68 ± 5.59	–	*t* = 0.35, *p* = 0.726
PANSS-G	46.70 ± 8.18	48.56 ± 6.49	–	*t* = −0.85, *p* = 0.400
PANSS-T	91.45 ± 15.80	92.68 ± 10.50	–	*t* = −0.31, *p* = 0.756
**COGNITIVE FUNCTION^a^**				
WCST-C	51.50 ± 7.95	49.64 ± 5.99	32.00 ± 10.35	*F* = 40.17, *p* <**0.001**
WCST-PE	3.93 ± 0.83	3.91 ± 0.81	4.53 ± 1.06	*F* = 3.80, *p* = **0.027**
VFT	17.71 ± 4.76	16.95 ± 6.63	23.21 ± 5.52	*F* = 10.01, *p* <**0.001**
TMT-A	57.92 ± 31.61	65.69 ± 44.48	34.69 ± 13.52	*F* = 8.68, *p* <**0.001**
TMT-B	163.10 ± 77.49	162.77 ± 90.41	71.82 ± 21.84	*F* = 20.36, *p* <**0.001**
DST-F	7.71 ± 1.38	7.45 ± 1.14	8.66 ± 1.26	*F* = 7.38, *p* = **0.001**
DST-B	5.21 ± 1.37	4.91 ± 1.38	6.50 ± 1.45	*F* = 10.24, *p* <**0.001**
Stroop-W	25.38 ± 10.89	23.15 ± 13.32	14.74 ± 4.13	*F* = 9.84, *p* <**0.001**
Stroop-C	26.39 ± 11.70	27.23 ± 13.63	15.92 ± 4.33	*F* = 12.44, *p* <**0.001**
Stroop-I	40.41 ± 25.73	38.90 ± 13.63	27.21 ± 8.62	*F* = 6.53, *p* = **0.002**

*PANSS-P, positive PANSS subscale score; PANSS-N, negative PANSS subscale score; PANSS-G, general psychopathology PANSS subscale score; PANSS-T, total PANSS-T score; WCST-C, Wisconsin Card Sorting Test categories; WCST-P, Wisconsin Card Sorting Test perseverative errors; VFT, Verbal Fluency Test; TMT-A, Trail Making Test-Part A; TMT-B, Trail Making Test-Part B; DST-F, Digit Span Tests Forward; DST-B, Digit Span Tests Backward; Stroop-W, Stroop Tests Words; Stroop-C, Stroop Tests Colors; Stroop-I, Stroop Tests Interference. Bold values indicate significant difference (P < 0.05)*.

a *36 patients (14 responders and 22 non-responders) and 38 controls were assessed cognitive function*.

One-way ANOVAs for cognitive function found that there are significant differences among three groups in all items (Table 1). Among them, however, WCST-PE did not pass Bonferroni testing (*p* > 0.05). Furthermore, *post-hoc* analyses for cognitive function demonstrated no significant differences in WCST-C (d = 1.86, *p* = 0.539), WCST-PE (d = 0.02, *p* = 0.952), VFT (d = 0.76, *p* = 0.700), TMT-part A (d = −7.77, *p* = 0.442), TMT-part B (d = 0.33, *p* = 0.988), DST-Forward (d = 0.26, *p* = 0.545), DST-Backward (d = 0.31, *p* = 0.529), Stroop-W (d = 2.23, *p* = 0.476), Stroop-C (d = 0.84, *p* = 0.797) and Stroop-I (d = 1.51, *p* = 0.765) between responder and non-responder patients groups.

### *COMT, 5-HTR2A* and *SLC6A4* mRNA expressions in non-responder and responder patients vs. healthy control subjects

**Figure 1:**
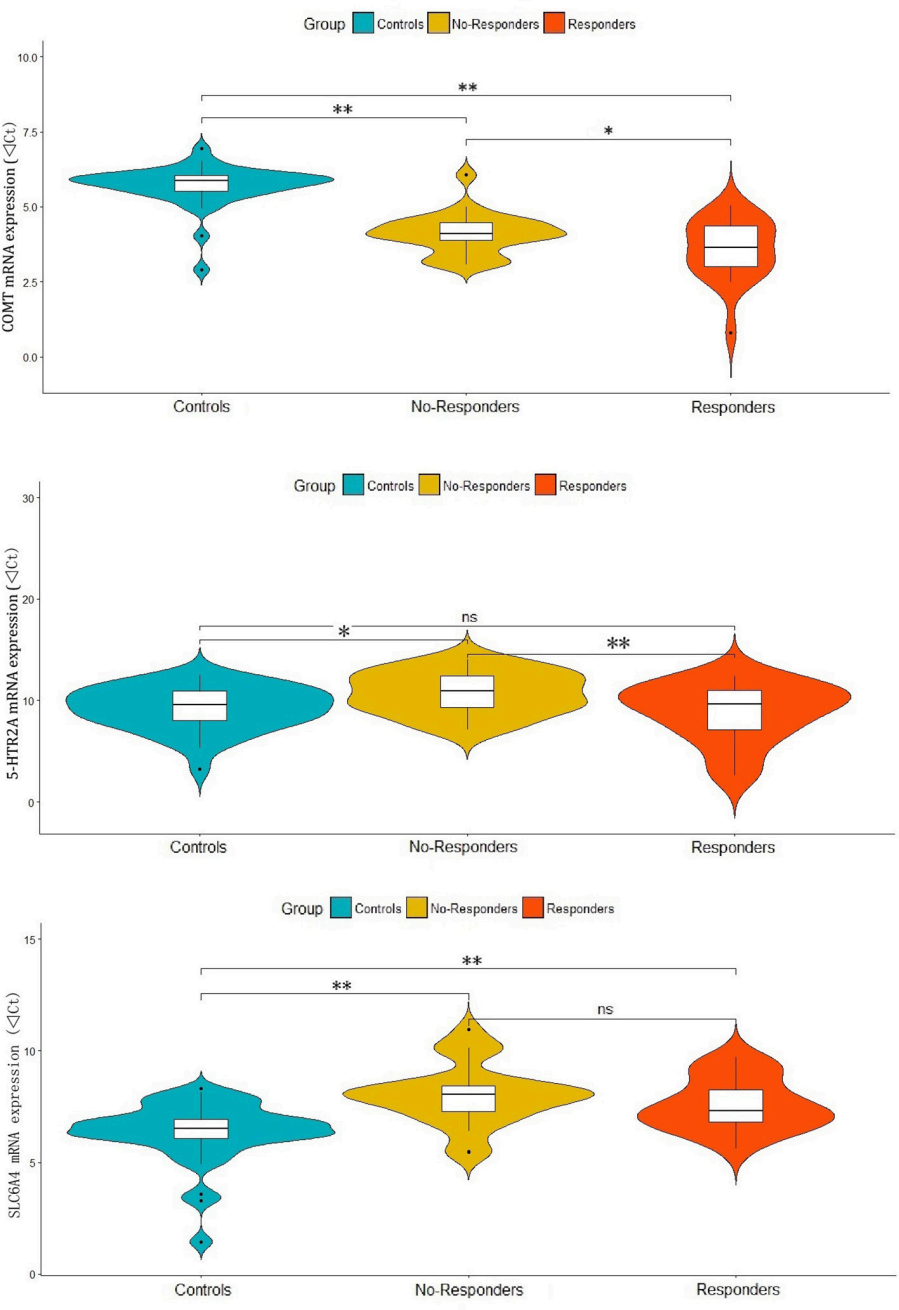
Differential expressions of COMT, 5-HTR2A and SLC6A4 in responder and non-responder of patients and healthy controls. **p-value < 0.01, *p-value < 0.05 and ns, no significance.

In the one-way ANOVAs, we found significant differences among responder, non-responder and control groups for the mRNA expressions of *COMT* (*F* = 61.05, *p* < 0.001), *5-HTR2A* (*F* = 4.60, *p* = 0.013) and *SLC6A4* (*F* = 13.73, *p* < 0.001). The *post-hoc* analyses showed that compared with controls, both responders and non-responders have significant higher *COMT* expression (d = 2.12, *p* < 0.001 and d = 1.60, *p* < 0.001) and lower *SLC6A4* expression (d = −1.23, *p* = 0.001 and d = −1.66, *P* < 0.001). While a significant lower level of *5-HTR2A* expression was observed in non-responders (d = −1.49, *p* = 0.01) when compared with controls, but not observed in responders (d = 0.40, *p* = 0.530). Interestingly, responders had significantly higher COMT (d = 0.52, *p* = 0.027) and *5-HTR2A* (d = 1.89, *p* = 0.007) expressions when compared with non-responders. However, there were no significant difference in *SLC6A4* expression (d = 0.43, *p* = 0.277) between responders and non-responders (Figure 1).

### Interaction of *COMT, 5-HTR2A*, and *SLC6A4* mRNA expressions in schizophrenia patients and controls

Partial correlation analyses showed significant positive correlations between *5-HTR2A* and *SLC6A4* mRNA expressions in both schizophrenia patients (r = 0.44, *p* = 0.004) and controls (r = 0.69, *p* < 0.001). These positive correlations remain significant after corrected with Bonferroni (*p* < 0.05). However, no significant correlation was observed among other genes (all *p* > 0.05).

### Association of *COMT, 5-HTR2A*, and *SLC6A4* mRNA expressions with baseline symptom severity, cognitive function, and cognitive improvement in schizophrenia patients

Stepwise multiple regression analyses found that no factor has a significant effect on the total PANSS score. In addition, analyses for cognitive function identified *5-HTR2A* as the influencing factor for VFT (β = −0.47, *p* = 0.003), Stroop-W (β = 0.42, *p* = 0.012), Stroop-C (β = 0.36, *p* = 0.033) and Stroop-I (β = 0.45, *p* = 0.006). Similarly, age was identified as a influencing factor for DST-Backward (β = −0.39, *p* = 0.018) and TMT-part B (β = 0.47, *p* = 0.004). In addition, stepwise multiple regression analyses for cognitive improvement over 8-week treatment suggested *SLC6A4* as a influencing factor for DST-Forward improvement (β = −0.45, *p* = 0.005), *5-HTR2A* as a influencing factor for Stroop-I improvement (β = 0.40, *p* = 0.017), and education level as a influencing factor for Stroop-C improvement (β = −0.46, *p* = 0.005). However, all of these findings did not pass Bonferroni testing.

### Antipsychotic effect on *COMT, 5-HTR2A*, and *SLC6A4* mRNA expression in schizophrenia patients

We conducted RMANOVAs to evaluate the gene expression changes over 8 weeks in responders and non-responders. Analyses revealed that there were no significant group effect and time effect on *COMT* (*p* = 0.069 and *p* = 0.520, respectively), *5-HTR2A* (*p* = 0.139 and *P* = 0.841, respectively) and *SLC6A4* (*p* = 0.966 and *p* = 0.163, respectively) mRNA expressions with no group by time interaction (*p* = 0.471, *p* = 0.053 and *p* = 0.111, respectively).

## Discussion

The present study has three major findings. (1) There are significantly higher *COMT* mRNA expression and lower *SLC6A4* mRNA expression in schizophrenia patients, and a significant lower *5-HTR2A* mRNA expression in non-responder patients when compared with healthy controls. (2) Responder patients have significantly higher levels of *COMT* and 5-HTR2A mRNA expressions than non-responder patients before antipsychotic treatment. (3) Antipsychotic treatment has no significant effect on the expressions of *COMT, 5-HTR2A* and S*LC6A4* mRNA over 8-week follow up.

*COMT* is a catabolic enzyme involved in the degradation of cortical dopamine and plays a critical role in modulating the activity of prefrontal circuitry. Previous genetic association studies suggested that individuals with high-activity variants have a higher risk for schizophrenia (16). These findings are consistent with our result that schizophrenia patients have a significantly increased *COMT* mRNA expression compared with controls. However, prior studies have reported inconsistent results regarding *COMT* mRNA expression in schizophrenia. For example, although many studies reported no evidence of altered *COMT* mRNA expression in schizophrenia (38–40), some studies demonstrated a lower *COMT* mRNA expression in patients (24, 41). One possible explanation for these discrepancies is that several factors such as demographics, medications, disease state, tissues, and expression assays might confound these results across different studies.

More interestingly, we found that the aberrant *COMT* mRNA expression in responder patients is more serious than that of in no-responder patients, indicating that patients with higher level of *COMT* mRNA were more likely to have a better antipsychotic response. This finding is consistent with the tonic-phasic dopamine hypothesis regarding the antipsychotic response (42). According to this hypothesis, the high activity of *COMT* enzyme can increase phasic and decrease tonic dopamine transmission subcortically and reduce dopamine concentrations in PFC, then lead to an increased D2–mediated dopamine transmission (42). As a result, individuals with higher activity of *COMT* enzyme will have a better response to D2-blocking agents such as risperidone.

A further major finding of this study is that non-responder patients have a significantly lower *5-HTR2A* mRNA expression when compared with responder patients. The serotonin 2A receptor is the most important excitatory receptor of serotonergic system and regulates serotonin transmission in the brain (3). In addition, the serotonin 2A receptor also interacts with dopamine systems and indirectly increases dopamine release in the PFC (11). As one of the main antipsychotic target, *5-HTR2A* has been suggested as an important player in antipsychotic treatment. Numerous pharmacogenetic studies have reported that *5-HTR2A* rs6314 T allele is associated with a poor treatment response to antipsychotic medicines (43–45). More recently, a study with converging evidence suggests rs6314 T allele significantly associated with decreased *5-HTR2A* mRNA in postmortem prefrontal cortex and HeLa cells (46). Taken together, these evidences indirectly support our finding that decreased *5-HTR2A* mRNA may predict a poor response to antipsychotic treatment.

The *SLC6A4* gene encodes serotonin transporter that plays a critical role in serotonergic neurotransmission by the reuptake of serotonin from the synaptic cleft. Our study found decreased *SLC6A4* mRNA expression in schizophrenia, which is consistent with a previous postmortem study(25), indicating that the reduced *SLC6A4* mRNA may implicate in the pathophysiological process of schizophrenia. Interestingly, we found a significant positive correlation between *5-HTR2A* and *SLC6A4* mRNA expressions in all subjects. This is similar to a prior study which suggested that interaction between variants of *5-HTR2A* and *SLC6A4* polymorphisms might be involved in the etiology of schizophrenia (47). Further evidence showed that serotonin transporter binding has been reported to be heavily colocalized with *5-HT2A* receptors in prefrontal regions (48), and the *HTR2A* gene appears to moderate serotonin transporter binding in key limbic regions (49). Taken together, these findings support that the serotonin transporter and *5-HT2A* receptor are biologically and functionally linked although the precise mechanism in schizophrenia is still unclear.

Finally, our findings suggest that there is no significant effect of antipsychotic treatment on *COMT, 5-HTR2A*, and *SLC6A4* mRNA expressions. Several previous studies found that there are prominent alterations in these genes expressions after antipsychotic treatment, but with exactly opposite effects (26, 41, 50). Furthermore, some studies reported these genes' expressions existing region-specific alterations along with antipsychotic treatment (51–53). Given that the relationships between antipsychotic drugs and dopaminergic/serotonergic genes expressions (*COMT, 5-HTR2A*, and *SLC6A4*) in schizophrenia remain inconclusive, it is necessary to elucidate the mechanisms of antipsychotic drugs implicated in dopaminergic/serotonergic genes expressions before any final conclusions to be made.

This study has some limitations. Firstly, our study investigated gene expression from a mixture of peripheral blood cells. Given that schizophrenia is one of brain disorders, there are evident limitations using peripheral blood cells to explore the disease pathophysiology although some studies have suggested blood shares significant gene expression similarities with brain tissues (54, 55). Secondly, there are potential eQTLs effects that may cofound our results; however we did not genotype the variants of targeted genes to preclude this confounding factor. Thirdly, the sample size in this study is relatively small, which might lead to insufficient statistical power to detect some true and slight differences. Finally, our study focused on three well-known candidate genes, it precludes the discovery of dopaminergic and serotonergic genes that may play important roles in disease pathophysiology and antipsychotic treatment. Therefore, our results should be cautiously interpreted with the consideration of these limitations.

In summary, dysregulated *COMT* and *SLC6A4* mRNA expressions in schizophrenia provides further evidence supporting that these two genes may implicate in the pathophysiology of schizophrenia. The higher *COMT* and *5-HTR2A* mRNA expressions in the responder patients indicate that these two genes mRNA may serve as predictors for antipsychotic treatment. However, the mechanisms of COMT and 5-HTR2A implicated in symptom improvement over antipsychotic treatment are still unknown, which deserve further investigation in the future.

## Ethics statement

This study was carried out with written informed consent from all subjec0ts. All subjects gave written informed consent in accordance with the Declaration of Helsinki. The protocol was approved by The Ethics Committee of the Second Xiangya Hospital of Central South University, China.

## Author contributions

JT, XC, MH, and XZ designed the study. MH, XZ, YH, HH, DW, JZ, HR, and LY collected the samples. ZL, YZ, XM acquired the data. ZL and YH analyzed the data and wrote the article. All authors have approved the submission.

### Conflict of interest statement

The authors declare that the research was conducted in the absence of any commercial or financial relationships that could be construed as a potential conflict of interest.
